# Physical Activity as a Determinant of Quality of Life in Working-Age People in Wrocław, Poland

**DOI:** 10.3390/ijerph15040623

**Published:** 2018-03-29

**Authors:** Daniel Puciato, Michał Rozpara, Zbigniew Borysiuk

**Affiliations:** 1 Faculty of Physical Education and Physiotherapy, Opole University of Technology, ul. Prószkowska 76, 45-758 Opole, Poland; z.borysiuk@po.opole.pl; 2 Faculty of Physical Education, The Jerzy Kukuczka Academy of Physical Education in Katowice, ul. Mikołowska 72, 40-065 Katowice, Poland; m.rozpara@awf.katowice.pl

**Keywords:** physical activity, quality of life, working-age

## Abstract

Regular physical activity can greatly contribute to the improvement of physical fitness and performance, reduction of the incidence risk of some occupational diseases, and as a consequence, to a general improvement of quality of life in terms of health status. The aim of the article was to assess relationships between the quality of life and physical activity of a working-age population. The study material comprised 4460 residents of the city of Wrocław, Poland (2129 men, 2331 women) aged 18–64 years. The study was a questionnaire survey using the International Physical Activity Questionnaire Short Form (IPAQ-SF) and The World Health Organization Quality of Life (WHOQOL-BREF) questionnaires. The highest levels of overall quality of life and its four particular domains (physical, psychological, social, and environmental), as well as perceived health conditions were found among the most physically active respondents. Furthermore, the odds of high assessment of perceived overall quality of life were shown to increase with the increasing levels of physical activity. Activities aimed at the improvement of the quality of life of working-age people should involve programs enhancing the development of physical activity.

## 1. Introduction

Health benefits of regular physical activity have been empirically well-documented. Earlier study results indicate that physical exercise can effectively contribute to the improvement of physical fitness [1,2] and the reduction of the risk of prevalence of such diseases as muscle atrophy [3], sarcopenia [4], osteoporosis [5], type 2 diabetes [6], obesity [7], arterial hypertension [8], coronary heart disease [9], and some types of cancer [10]. Researchers also noted positive effects of physical activity on mental health, such as stress alleviation [11], improvement of perceived health condition and self-esteem [12], reduction of anxiety and depression levels [13], or improvement of sleep sufficiency [14].

Undertaking physical activity by people of working age is a significant problem. Their engagement in physical exercises of appropriate frequency, duration, and intensity can not only contribute to the prevention of diseases and maintaining good psychophysical fitness [15], but it can also ensure full recovery after diseases, injuries, or fatigue [16]. As a consequence, engagement in physical activity allows working-age individuals to successfully fulfill their social, professional, family, or sexual functions [17,18,19] and, thus, it greatly affects their perceived quality of life.

Relationships between quality of life and physical activity have been thoroughly studied in the elderly [20,21,22], also in our earlier research [23], or in patients with dementia [24], coronary heart disease [25], osteoporosis [26], and renal diseases [27]. Some authors have indicated that physically active individuals perceived certain components of their quality of life better than their non-active counterparts. However, an improvement in perceived quality of life was observed only in respondents whose levels of physical activity were sufficiently high [28]. This is highly significant since some researchers noted that a high percent of adult Poles do not meet physical activity standards, and that the duration of physical exercise was shown to decrease with age [29,30]. The aim of the present study was to identify the relationships between the overall quality of life and its particular domains (physical, psychological, social, and environmental), and perceived health condition and physical activity levels in working-age residents of the city of Wrocław, Poland.

The main hypothesis of the study predicted there would be correlations between quality of life and physical activity, i.e., that high physical activity levels would have a positive impact on the quality of life and perceived health status of working-age respondents from Wrocław. It was also assumed that the respondents’ sex can be a modifier of these hypothetical correlations.

## 2. Methods

The study was carried out in 2014 and 2015 in Wrocław, Poland. The research project had been given a positive opinion by the Commission of Bioethics of the University School of Physical Education in Wroclaw. The research material comprised 4460 residents (2129 men; 2331 women) aged 18–64 years. Ethical Approval: This research project has been granted a positive opinion by the Commission of Bioethics of the University School of Physical Education in Wroclaw.

The men’s mean body height was 179.1 ± 8.1 cm, body mass 81.6 ± 12.3 kg, and BMI 25.4 ± 3.3 kg/m^2^. The women’s mean body height was 166.0 ± 7.0 cm, body mass 63.7 ± 9.9 kg, and BMI 23.2 ± 3.6 kg/m^2^. Among the studied Wrocław residents 39% had a primary and vocational education, 36% secondary education, and 25% had a college degree. The highest percentage of residents were professional workers (44% white-collar workers, 26% manual workers), the others were students (school pupils and college students), and non-workers (housekeepers and unemployed), 14% and 15%, respectively. The minimal sample size (n = 3955) representative of all working-age Wrocław residents was calculated following the formula [31]:

$$n=\frac{N}{1+\frac{e^{2}(N-1)}{u_{\alpha }^{2}\mathrm{pq}}}$$

where N is the number of Wrocław residents on 31 December 2013 (N = 632,067), p is the fraction of working-age Wrocław residents on 31 December 2013 (p = 0.63), q is the constant calculated as 1 − p (q = 0.37), e is the expected estimation error of p (e = 1.5%), and u_α = 0.05_ is the confidence interval 1 − α (u_α=0.05_ = 1.96). Fifteen percent more questionnaires were collected, accounting for a possibility of errors made by respondents. After rejecting incomplete or incorrectly-completed questionnaires, the number of questionnaires subject to analysis was 4460.

The sample selection was random using a three-level stratification. First, using a random number table, ten residential areas were selected from all alphabetically ordered Wrocław areas. Next, three streets from each selected residential area were chosen, whose residents were asked to fill in the questionnaires. The number of respondents from particular residential areas was proportionate to the number of residents of these areas. The respondents were all informed about the purpose and the course of the study, and they expressed their written consent to participate.

The main method used in the study was the diagnostic questionnaire survey. Respondents’ habitual physical activity was assessed with the International Physical Activity Questionnaire Short Version (IPAQ-SF). Following IPAQ guidelines the respondents were classified as engaged into vigorous physical activity, moderate physical activity, and low physical activity [32].

Respondents’ quality of life was assessed with the World Health Organization Quality of Life (WHOQOL-BREF) questionnaire [33]. The reliability and accuracy of the WHOQOL-BREF tool was confirmed by other authors [34,35]. Overall quality of life was additionally expressed on a nominal scale. The results of overall quality of life assessment expressed on an interval scale (1–5 points) were then converted into sten scores (1–10 tens) using the formula: STEN = z(SD) + M, where z was the standardized score on the original interval scale, SD = 2.0, M = 5.5, for example STEN = 1.3 (2.0) + 5.5 = 8.0 [36]. The respondents were then grouped into three quality of life ranges: <6 stens—low; 6 stens—medium; and >6 stens—high.

The number and percentage of respondents were calculated for overall quality of life and physical activity level in different categories. Arithmetic means, standard deviations, and mean ranks were calculated for overall quality of life and its particular domains, as well as for perceived health condition. The chi-square goodness of fit test was used to assess the significance of differences between the numbers of respondents classified according to their levels of quality of life and classified according to their physical activity level.

Differences in the average level of quality of life in groups of people due to their physical activity levels were assessed with ANOVA (means, Kruskal-Wallis test, Dunn’s post hoc multiple comparison test) and multinomial logistic regression analysis. The level of statistical significance was set at α < 0.05. The impact of physical activity levels on respondents’ quality of life was estimated using Eta-squared (η^2^). The effect sizes were categorized into small η^2^ = 0.01, medium η^2^ = 0.06, and large η^2^ = 0.14. All statistical calculations were made with the use of IBM SPSS Statistics 20 (Armonk, NY, USA).

## 3. Results

Among the working-age men from Wrocław the highest percentage were respondents assessing their quality of life as average (47.7%), followed by those assessing it as low (34.6%), and as high (17.8%). Similar percentages were found among the female respondents: average—48.2%, low—37.9%, and high—13.9%. The differences in the number of respondents in groups assessing their quality of life differently were statistically significant both among men and women (*p* < 0.001) (Table 1).

Among the men the highest percentage were respondents with a high level (45.6%), followed by respondents with a moderate level (34.3%), and with a low level (20.1%) of physical activity. In women, the largest group were those with a moderate physical activity level (43.5%), followed by those with a high level (40.2%), and a low level (16.3%). The numbers of respondents in groups according to their levels of physical activity differed significantly (*p* < 0.001) among men and women (Table 1). The mean level of physical activity among the residents from Wrocław was, thus, higher than indicated in earlier studies of residents of Katowice [29], Szczecin [21], and Warszawa [30].

Statistically significant differences were noted between mean quality of life indices in men from groups of different levels of physical activity (H = 18.9, *p* < 0.001). The men with high and low levels of physical activity had the highest levels of overall quality of life (3.8 pts.), whereas the men with the moderate physical activity levels had the lowest overall quality of life (3.6 pts.). The differences in self-perception of quality of life among respondents with high and moderate physical activity levels were statistically significant (*p* < 0.001). Such differences were not found between groups of respondents with high and low levels of physical activity, and with moderate and low levels of physical activity (Table 2).

The mean perception of health condition was the highest in the most active men (3.7 ± 0.9 pts.), followed by men with low levels of physical activity (3.5 ± 0.9 pts.), and men with moderate levels of physical activity (3.4 ± 1.1 pts.). Differences in perceived health condition in groups with different physical activity levels were statistically significant (H = 57.2, *p* < 0.001). Post hoc comparisons between groups with different PA levels revealed statistically significant differences in perceived health condition between respondents with high and moderate PA levels, and high and low PA levels (*p* < 0.001) (Table 2).

The male respondents with different physical activity levels also differed significantly in their assessments of four quality of life domains: physical, psychological, social, and environmental. Men who were the most physically active had also the highest scores in particular quality of life domains. The lowest quality of life in its somatic domain was found in the least physically active male residents from Wrocław. The men with moderate levels of physical activity had the lowest levels of quality of life in the psychological, social and environmental domains. The noted differences were statistically significant (*p* < 0.001). Inter-group Dunn’s post hoc multiple comparison test results showed statistically significant differences (*p* < 0.001) in the social domain between groups with low and high, and moderate and high physical activity levels, and in the physical, psychological, and environmental domains, also between the groups with low and moderate physical activity levels. The effect size of physical activity on quality of life domains in the studied group of male residents was between η^2^ 0.01 and 0.04 (Table 2).

The female respondents with high levels of physical activity featured the highest overall quality of life (3.8 ± 0.8 pts.). Lower overall quality of life assessments (3.7 ± 0.8 pts.) were made by women with low physical activity levels. The women with a moderate level of physical activity had the lowest overall quality of life (3.6 ± 0.8 pts.). The mean ranks of overall quality of life among the women with different levels of physical activity differed significantly from each other (H = 29.1, *p* < 0.001). Among the studied female residents there were significant differences in perceived quality of life between women with high and low levels of physical activity (*p* < 0.01), and between women with high and moderate levels of physical activity (*p* < 0.001) (Table 3).

The highest mean perceived health condition was assessed by women with the highest physical activity levels (3.6 ± 0.9 pts.). PHC was lower in women with low physical activity levels (3.4 ± 1.1 pts.) and with moderate physical activity levels (3.4 ± 1.0 pts.). The differences in perceived health condition between groups with different physical activity levels were statistically significant (H = 28.1, *p* < 0.001). The post hoc comparisons between the groups of respondents at different levels of physical activity revealed statistically significant differences in perceived health condition between women with high and moderate levels of physical activity, and between women with high and low levels of physical activity (Table 3).

The mean assessments of particular domains of quality of life differed significantly and were the highest in women with a high level of physical activity. In the psychological and social domains the lowest quality of life was noted in women with the lowest levels of physical activity; and in the physical and environmental domains, in women with a moderate level of physical activity. In the groups of female respondents with different levels of physical activity the mean rank values of all quality of life domains differed significantly (*p* < 0.05). The post hoc inter-group comparisons revealed statistically significant differences between groups with low and moderate physical activity levels, and between groups with moderate and high physical activity levels in the physical domain; between groups with low and high, low and moderate, and moderate and high physical activity levels in the social domain; between groups with low and high physical activity levels in the psychological domains; and between groups with moderate and high physical activity levels in the environmental domain. The size of effect of physical activity on quality of life domains among the studied female residents amounted to η^2^ = 0.01 (Table 3).

Table 4 and Table 5 present models of multinomial logistic regression analysis illustrating relationships between overall quality of life with the levels of physical activity of working-age residents of Wrocław. The reference category for the trichotomous variable of quality of life was the low level of quality of life. The goodness of fit of both models (*χ*^2^ = 26.4, *χ*^2^ = 55.9) and their probability (*p* < 0.001) indicate that these models differed significantly from models containing only intercepts, and that the level of physical activity also significantly affected the quality of life of men and women under study.

In the group of working-age men the odds of assessing their quality of life as average, and not low, were (1.00 − 0.67) × 100% = 33% lower in respondents with a moderate level of physical activity than in respondents with a high level of physical activity (1.00 is the odds ratio in the group of respondents with a high level of physical activity, regarded as a reference category for the intercept of the model). The odds that the male respondents assessed their quality of life as high, and not low, were 41% lower in respondents with low physical activity levels than in respondents with a high level of physical activity, and 35% lower in men with a moderate level of physical activity than in men with high physical activity levels (Table 4).

The odds of assessing their quality of life as average, and not as low, by the female respondents were nearly two times lower in women with low levels of physical activity (OR = 0.51) (odds ratio), than in respondents with high levels of physical activity; and 15% lower in women with moderate levels of physical activity than in women with high physical activity levels. The odds of assessing their quality of life as high, and not as low, was 56% lower in female respondents with moderate levels of physical activity than in respondents with a high level of physical activity. In the case of female Wrocław residents with low levels of physical activity these odds were 37% lower than in women with high levels of physical activity (Table 5).

## 4. Discussion

The results of the present study revealed positive correlations between quality of life and physical activity levels in working-age people. The highest overall quality of life, perceived health condition, and quality of life in its particular domains: physical, psychological, social, and environmental, were noted in those working-age men and women from Wrocław whose level of physical activity was high. Additionally, the odds of assessing one’s overall quality of life as average and not as low, and as high and not as low, was the highest in respondents with the highest physical activity levels. These results show clearly that perceived quality of life was significantly associated by the high level of physical activity in respondents of both sexes. Following the IPAQ guidelines, respondents with high physical activity levels were defined as those who performed vigorous physical exercise for three or more days a week at the caloric cost of at least 1500 MET min/week, or who performed any combination of low-, moderate-, or high-intensity activities for seven days a week a caloric cost of at least 3000 MET min/week.

Additionally, Brown et al. [37] found a significant improvement in perceived quality of life in respondents who undertook moderate-level physical activities for at least 30 min a day for five days a week; or vigorous physical activities for at least 20 min a day for three days a week. Guimarães and Baptista [38] noted that undertaking 60 min of moderate physical activity a day was significantly positively correlated with the quality of life of women aged 45–59 years. Leino-Arjas et al. [39], however, found that only regular physical activity of high intensity had a positive impact on perceived quality of life.

A significantly higher perceived overall quality of life among individuals with a high level of physical activity than among non-training control was also observed by Ramirez-Campillo et al. [40]. Positive correlations between overall quality of life and the duration of undertaken physical exercises were also indicated by Anokye et al. [41], Rosenkranz et al. [42], and Kolt et al. [43].

Krzepota et al. [21], Quehenberger et al. [22], and Kim et al. [44] found positive correlations between perceived health condition and the level of physical activity. This can be justified by the proven positive impact of physical exercise of appropriate duration, frequency, and intensity on health condition in adults [1,2]. Perceived health condition, however, does not only depend on one’s objective health status, but it is also determined by psychological and cultural factors, such as one’s lifestyle or socioeconomic status. For example, Trentini et al. [45] revealed that individuals with a higher education, who belong to the middle or high social classes, and do not suffer from depression or low moods, perceived their health condition much better.

The results of the present study of Wrocław residents aged 18–65 years also confirmed the positive effects of physical activity on the physical domain of quality of life noted in some earlier works [21,46,47]. Lifestyle, including physical activity, is not only a significant determinant of human health [15]. Positive correlations between physical activity and physical fitness [2,15] and performance [1] were well documented. Sporndly-Nees et al. [48] noted a positive impact of physical activity on patients with insomnia. Physical exercises can also have a significant preventive effect on incident insufficient sleep and can effectively maintain sleep sufficiency in healthy individuals [14].

Krzepota el al. [21] and Chai et al. [28] also noted positive correlations between physical activity and the psychological domain of quality of life. There have been confirmed relationships between physical activity and such factors as optimism and joy of life [49], visual attention [50], self-esteem [51], and reduced anxiety or depression [52].

A higher assessment of the social domain of the quality of life of physically active individuals was also noted by Guimarães and Baptista [38]. Researchers also described the positive effects of physical activity on the proper development of interpersonal relations [53] and sexual life [19].

The least studied were relationships between physical activity and the environmental domain of the quality of life; however, authors showed that health-enhancing physical exercises of appropriate duration, frequency, and intensity were correlated with such quality of life components as good environmental features [54], socioeconomic conditions [55], or leisure pursuits [56].

Among the studied Wrocław residents sex was not a determinant of correlations between physical activity and quality of life. Thus, the results of Huang-tz et al. [57], who showed a significant impact of the male sex, were not confirmed. It might be explained by an observation that for, an increasing number of modern women, physical activity is becoming a key lifestyle component. This can be confirmed by relatively similar levels of physical activity of the male and female residents in the present study, contrary to the results of some earlier research which had indicated significantly higher levels of physical activity among men [29,30,55]. The lack of intersex differences can be also explained by the respondents’ place of residence. Due to dynamic social changes in large urban areas the lifestyle differences between men and women can be insignificant. Large cities have a well-developed sport infrastructure and offer multiple recreational possibilities for women. Furthermore, the studied women were better educated than the men, which could have also influenced the aforementioned observation. A higher level of education is often associated with a higher level of knowledge about the importance of physical activity for health and quality of life. Positive correlations between physical activity and quality of life in women were also found by Krzepota et al. [21], Guimarães and Baptista [38], and Gomez et al. [58].

The present study has some strengths and weaknesses. Its main advantage is the broad age range of respondents (18–64 years) in comparison with many earlier studies. Few similar research studies have been published on Polish or Central European populations, and studies of possible correlations between physical activity and quality of life in respondents divided according to their sex have also been very rare. The applied data-gathering methodology is novel, in particular, in reference to the overall quality of life indices.

One drawback of the study is the limitation of the study population to a single city. Prospective research in this field should cover the whole of Poland, and even other countries of Central Europe. A certain weakness of the study is the application of the short version of the IPAQ. In the future researchers should use methods allowing the measurements of physical activity in different spheres of life, e.g., during leisure time, at work, while getting from place to place, or while performing domestic chores, since the strength and directions of correlations of physical activity with quality of life can vary, depending on these areas [59]. Another shortcoming of the study is the analysis of the whole Wrocław working-age population. It is suggested that prospective research should focus on the effects on the physical activity of quality of life in groups of respondents divided according to age, education level, occupation, or socio-economic status.

## 5. Conclusions

The study results confirm the existence of positive correlations between physical activity and quality of life. For working-age individuals physical activity of appropriate duration, frequency, and intensity is a significant determinant of their overall quality of life and its particular domains: physical, psychological, social, and environmental. Therefore the main hypothesis of the present study assuming the positive effects of physical activity on the quality of life and perceived health status of working-age respondents from Wrocław has been confirmed. A low impact of physical activity on quality of life can result from the complexity of the notion of quality of life itself. According to Borys [60] the number of potential variables affecting perceived quality of life is extensive. However, the hypothesis about sex as a determinant of correlations between quality of life and physical activity must be refuted as these correlations were, in fact, statistically significant in both men and women.

The results of the present study can be also applied in practice. Since improvement of quality of life is one of the main strategic development goals of cities or regions, raising physical activity levels in municipal communities should also be accounted for in these strategies. The improvement of physical activity in society should be also a key area of all public health programs and subject to such incentives by public authorities as developing sport infrastructure, lowering indirect taxes on sport and recreation equipment, or conducting community campaigns promoting physical activity. An important role for implementation of such tasks, especially in relation to working-age people, can be fulfilled by employers, who could organize recreational activities during work breaks, co-fund leisure sport activities for employees, or provide leisure facilities or bicycle storage rooms. Such activities may not only be beneficial to the employees, but by improving their health status and work efficiency, they can also enhance company business output.

## Figures and Tables

**Table 1:** Number and percentage of respondents in groups with different levels of overall quality of life and physical activity.

Variable	Category	Group	Number	Percent	*χ*^2^	*p*-Value
Overall quality of life	LQOL	Men	736	34.6	287.4	<0.001
	AQOL		1015	47.7		
	HQOL		378	17.8		
	LQOL	Women	884	37.9	435.0	<0.001
	AQOL		1124	48.2		
	HQOL		323	13.9		
Physical activity level	LPAL	Men	428	20.1	207.9	<0.001
	MPAL		731	34.3		
	HPAL		970	45.6		
	LPAL	Women	379	16.3	309.5	<0.001
	MPAL		1014	43.5		
	HPAL		938	40.2		

Notes: *χ*^2^—chi-square goodness of fit test, *p*—chi-square goodness of fit test probability value. Abbreviations: LQOL—low quality of life, AQOL—average quality of life, HQOL—high quality of life; LPAL—low physical activity level, MPAL—moderate physical activity level, HPAL—high physical activity level.

**Table 2:** Quality of life in groups of men with different levels of physical activity.

Quality of Life	Physical Activity Level	$\bar{x}$ ± SD	$\bar{R}$	H	*p*-Value	η^2^	Multiple Comparisons Mean Ranks Probability Value		
							LPAL vs. MPAL	LPAL vs. HPAL	MPAL vs. HPAL
OQOL	LPAL	3.8 ± 0.7	1053.6	18.9	<0.001		≥0.05	≥0.05	<0.001
	MPAL	3.6 ± 1.0	998.8			0.01			
	HPAL	3.8 ± 0.9	1119.9						
PHC	LPAL	3.5 ± 0.9	1021.0	57.2	<0.001		≥0.05	<0.001	<0.001
	MPAL	3.4 ± 1.1	958.3			0.03			
	HPAL	3.7 ± 0.9	1164.9						
PHYD	LPAL	12.5 ± 1.8	926.4	41.4	<0.001		<0.05	<0.001	<0.01
	MPAL	12.8 ± 1.5	1036.5			0.02			
	HPAL	13.1 ± 1.7	1147.6						
PSYD	LPAL	14.2 ± 1.7	1065.9	45.5	<0.001		<0.01	<0.05	<0.001
	MPAL	13.8 ± 1.9	949.8			0.02			
	HPAL	14.4 ± 1.8	1151.4						
SD	LPAL	15.3 ± 2.2	1035.1	31.0	<0.001		≥0.05	<0.01	<0.001
	MPAL	15.0 ± 2.6	980.7			0.01			
	HPAL	15.6 ± 2.4	1141.7						
ED	LPAL	13.5 ± 2.2	1025.0	79.9	<0.001		<0.05	<0.001	<0.001
	MPAL	13.2 ± 2.4	924.2			0.04			
	HPAL	14.1 ± 2.1	1188.7						

Notes: $\bar{x}$—mean, SD—standard deviation, $\bar{R}$—mean ranks, H—Kruskal-Wallis test, *p*—Kruskal-Wallis test probability value, η^2^—Eta-squared. Abbreviations: LPAL—low physical activity level, MPAL—moderate physical activity level, HPAL—high physical activity level, OQOL—overall quality of life, PHC—perceived health condition, PHYD—physical domain, PSYD—psychological domain, SD—social domain, ED—environmental domain.

**Table 3:** Quality of life in groups of women with different levels of physical activity.

Qualityof Life	Physical Activity Level	$\bar{x}$ ± SD	$\bar{R}$	H	*p*-Value	η^2^	Multiple Comparisons Mean Ranks Probability Value		
							LPAL vs. MPAL	LPAL vs. HPAL	MPAL vs. HPAL
OQOL	LPAL	3.7 ± 0.8	1105.9	29.1	<0.001		≥0.05	<0.01	<0.001
	MPAL	3.6 ± 0.8	1110.3			0.01			
	HPAL	3.8 ± 0.8	1250.6						
PHC	LPAL	3.4 ± 1.1	1140.7	28.1	<0.001		≥0.05	<0.05	<0.001
	MPAL	3.4 ± 1.0	1099.0			0.01			
	HPAL	3.6 ± 0.9	1248.6						
PHYD	LPAL	12.5 ± 1.8	1206.2	14.3	<0.01		<0.05	≥0.05	<0.01
	MPAL	12.3 ± 1.7	1106.3			0.01			
	HPAL	12.5 ± 1.8	1214.2						
PSYD	LPAL	13.6 ± 2.0	1102.4	7.4	<0.05		≥0.05	<0.05	≥0.05
	MPAL	13.7 ± 2.1	1151.9			<0.01			
	HPAL	13.8 ± 2.0	1207.0						
SD	LPAL	14.5 ± 3.0	1028.9	26.1	<0.001		<0.01	<0.001	<0.05
	MPAL	15.0 ± 2.7	1155.2			0.01			
	HPAL	15.3 ± 2.6	1233.1						
ED	LPAL	13.1 ± 2.4	1147.0	18.3	<0.001		≥0.05	≥0.05	<0.001
	MPAL	12.8 ± 2.3	1107.9			0.01			
	HPAL	13.3 ± 2.7	1236.5						

Notes: $\bar{x}$—mean, SD—standard deviation, $\bar{R}$—mean ranks, H—Kruskal-Wallis test, *p*—Kruskal-Wallis test probability value, η^2^—Eta-squared. Abbreviations: LPAL—low physical activity level, MPAL—moderate physical activity level, HPAL—high physical activity level, OQOL—overall quality of life, PHC—perceived health condition, PHYD—physical domain, PSYD—psychological domain, SD—social domain, ED—environmental domain.

**Table 4:** Multinomial logistic regression analysis parameters of the dependent variable: overall quality of life in studied men.

Overall Quality of Life	Parameter	*β*	SE	Wald *χ*^2^	*p*-Value	OR	OR 95%CI	
							LL	UL
AQOL	Intercept	0.46	0.07	38.9	<0.001			
	LPAL vs. HPAL	0.00	0.13	0.0	≥0.05	1.00	0.78	1.29
	MPAL vs. HPAL	−0.40	0.11	12.9	<0.001	0.67	0.54	0.84
HQOL	Intercept	−0.42	0.09	20.7	<0.001			
	LPAL vs. HPAL	−0.53	0.18	8.4	<0.01	0.59	0.41	0.84
	MPAL vs. HPAL	−0.44	0.14	9.5	<0.01	0.65	0.49	0.85

Notes: The reference category for the dependent variable is LQOL, *χ*^2^ = 26.4, *p* < 0.001, *β*—assessment value of model parameters, SE—asymptotic standard error *β*, Wald *χ*^2^—parameter significance, *p*—Wald χ^2^ probability value. Abbreviations: OR—odds ratio, CI—confidence interval, LL—lower limit, UL—upper limit, AQOL—average quality of life, HQOL—high quality of life, LQOL—low quality of life, LPAL—low physical activity level, MPAL—moderate physical activity level, HPAL—high physical activity level.

**Table 5:** Multinomial logistic regression analysis parameters of the dependent variable: overall quality of life in studied women.

Overall Quality of Life	Parameter	*β*	SE	Wald *χ*^2^	*p*-Value	OR	OR 95%CI	
							LL	UL
AQOL	Intercept	0.42	0.07	32.7	<0.001			
	LPAL vs. HPAL	−0.67	0.14	24.9	<0.001	0.51	0.39	0.66
	MPAL vs. HPAL	−0.16	0.10	2.5	≥0.05	0.85	0.70	1.04
HQOL	Intercept	−0.61	0.10	40.3	<0.001			
	LPAL vs. HPAL	−0.47	0.18	6.9	<0.01	0.63	0.44	0.89
	MPAL vs. HPAL	−0.81	0.15	29.8	<0.001	0.44	0.33	0.59

Notes: The reference category for the dependent variable is LQOL, *χ*^2^ = 55.7; *p* < 0.001, *β*—assessment value of model parameters, SE—asymptotic standard error *β*, Wald *χ*^2^—parameter significance, *p*—Wald χ^2^ probability value. Abbreviations: OR—odds ratio, CI—confidence interval, LL—lower limit, UL—upper limit, AQOL—average quality of life, HQOL—high quality of life, LQOL—low quality of life, LPAL—low physical activity level, MPAL—moderate physical activity level, HPAL—high physical activity level.
